# From High Intellectual Potential to Asperger Syndrome: Evidence for Differences and a Fundamental Overlap—A Systematic Review

**DOI:** 10.3389/fpsyg.2016.01605

**Published:** 2016-10-20

**Authors:** Aurélie Boschi, Pascale Planche, Cherhazad Hemimou, Caroline Demily, Laurence Vaivre-Douret

**Affiliations:** ^1^Paris Descartes University, Sorbonne Paris CitéParis, France; ^2^Institut National de la Santé et de la Recherche Médicale UMR 1018-CESP, Paris-Saclay-Paris Sud University, UVSQParis, France; ^3^Child Psychiatry Department, Necker-Enfants Malades University HospitalParis, France; ^4^CREAD (EA3875), Psychology Department, Bretagne Occidentale UniversityBrest, France; ^5^GénoPsy, Center for Diagnosis and Management of Genetic Psychiatric Disorders, Le Vinatier Hospital and EDR-Psy Team (Centre National de la Recherche Scientifique and Lyon 1 Claude Bernard University)Lyon, France; ^6^Department of Paediatrics, Child Development, Cochin-Port Royal University HospitalParis, France; ^7^IMAGINE Institute, Necker-Enfants Malades University HospitalParis, France

**Keywords:** Autism Spectrum Disorder, giftedness, asperger syndrome, high functioning autism, fundamental overlap, developmental trajectories

## Abstract

**Background:** An increasing number of clinicians point to similar clinical features between *some* children with High Intellectual Potential (HIP or “Giftedness” = Total IQ > 2 *SD*), and children with Autism Spectrum Disorder (ASD) without intellectual or language delay, formerly diagnosed with Asperger Syndrome. Some of these common features are social interaction impairments, special interests, and in some cases high-verbal abilities. The aim of this article is to determine whether these similarities exist at more fundamental levels, other than clinical, and to explore the literature in order to provide empirical support for an overlap between ASD and HIP.

**Method:** First, comparative studies between ASD and HIP children were sought. Because of a lack of data, the respective characteristics of ASD and HIP subjects were explored by a cross-sectional review of different areas of research. Emphasis was placed on psychometric and cognitive evaluations, experimental and developmental assessments, and neurobiological research, following a “bottom-up” procedure.

**Results:** This review highlights the existence of similarities in the neurocognitive, developmental and neurobiological domains between these profiles, which require further study. In addition, the conclusions of several studies show that there are differences between HIP children with a homogeneous Intellectual Quotient profile and children with a heterogeneous Intellectual Quotient profile.

**Conclusion:** HIP seems to cover different developmental profiles, one of which might share features with ASD. A new line of investigation providing a possible starting-point for future research is proposed. Its implications, interesting from both clinical and research perspectives, are discussed.

## Introduction

For some years, an increasing number of clinicians have signaled the difficulty in distinguishing, *in some cases*, high-functioning children with Autism Spectrum Disorder (ASD) from “gifted” children, but this question remains controversial and very little documented.

According to the Diagnostic and Statistical Manual of mental disorders fifth edition (DSM-5) (American Psychiatric Association, 2013), the diagnosis of ASD is based on two main symptom categories (criteria A and B), which can occur over three levels of severity: “criterion A: Persistent deficits in social communication and social interaction across multiple contexts,” “criterion B: Restricted, repetitive patterns of behavior, interests, or activities.” Criterion C specifies that these symptoms must be present in the early developmental period but may not become fully manifest until social demands exceed limited capabilities, or they may be masked by learned strategies in later life.

In the field of “giftedness,” four cases can be identified, sometimes interrelated, in which this term is used (Subotnik et al., 2011), namely to describe: (1) high academic achievers; (2) individuals who score at least 2 Standard Deviations (*SD*) above the average on intellectual tests (Full Scale Intellectual Quotient of 130 and more on the Wechsler Scales, widely used); (3) individuals exhibiting outstanding talent in one or more domains of ability and (4) a particular “profile” of individuals with high intellectual ability who also have socio-emotional specificities. These definitions of giftedness are based on different conceptions of the phenomenological reality that this term evokes, and on different models of intelligence. In the first case, giftedness is about academic achievement. In the second, it reflects high level of *intellectual* abilities, which are not a guarantee of academic achievement, while the third case refers to the development of natural abilities that depend on regular training, in any domain of general intelligence, based on pluralist models of intelligence. Finally, the last conception of giftedness primarily derives from clinical practice.

In this article, the term of “giftedness” will be intentionally avoided because of these conceptual disagreements, and the phrase “High Intellectual Potential” (HIP) will be preferred. HIP refers to individuals who obtain a Full Scale Intellectual Quotient (FSIQ) score of 130 or more on the Wechsler Scales, which explains the use of the term “intellectual.” Indeed, the “High Potential” explored in this article concerns the analytical aspects of intelligence, or Sternberg's “componential intelligence” (1985), measured by cognitive efficiency tests, which constitutes a particular domain of general intelligence.

From a clinical viewpoint, some children with HIP present difficulties associated with their high IQ (Pfeiffer, 2009), and sometimes fail to achieve in school for different reasons (Lupart and Pyryt, 1996). This observation generates conflicting positions. While there are HIP children without any difficulties, those who consult often have associated disorders. Most of the time, they also present considerable discrepancies across the factorial indices on the Wechsler Intelligence Scale for Children 4th edition—or WISC-IV—(Wechsler, 2003). Among these “consulting” children with HIP, clinicians have noted that some present common characteristics or clinical features with children with ASD without language or intellectual delay (Neihart, 2000; Liratni and Pry, 2011; Guénolé et al., 2013a; Doobay et al., 2014). These features are: social interaction impairments, emotional maladjustment, pedantic use of language, excessive focus on special interests, specific sensorial characteristics, withdrawal into abstraction (imaginary and/or intellectual) or attention deficits, and general clumsiness or recognized praxis disorders. Of course, these features do not systematically appear together and could have different origins depending on whether the child belongs to the autism spectrum or presents HIP. For example, some HIP children are socially isolated because they encounter problems meeting intellectual peers. Alongside, some children who have received a clear diagnosis of ASD also show high cognitive abilities attested by intelligence tests. Obviously, HIP and ASD are not diagnostically mutually exclusive. In this case, clinicians sometimes evoke a “twice-exceptional” condition (association between a disability and high ability/giftedness). However, a large proportion of HIP children share certain clinical signs with ASD without fully meeting the ASD diagnostic criteria. Thus, the question remains: how can we understand this clinical proximity between a certain form of HIP and ASD without language delay?

A growing number of clinicians consider that an overlap exists between ASD and HIP, and emphasize the difficulty in assessing the presence of ASD in a child with a high Intellectual Quotient (IQ) (Little, 2002; Lovecky, 2003; Assouline et al., 2009). This potential relationship between high intellectual abilities and ASD is not a novelty. Indeed, Asperger (1944) and Kanner (1943) both noted the frequent occurrence of individuals with intellectual activity or work in the families of their autistic patients.

The question of an overlap between ASD and HIP also reopens discussions on the distinction between High Functioning Autism (HFA) and Asperger Syndrome (AS) (Howlin, 2003; Macintosh and Dissanayake, 2004; Matson and Boisjoli, 2008; Kaland, 2011). In fact, according to the DSM-IV-TR, the main criteria enabling differentiation of Autistic Disorder [F84.0] without intellectual deficiency (or HFA) from AS [F84.5] were the absence of the criterion “Qualitative impairments in communication” and the absence of a language delay in AS. In the DSM-5 however, language development is no longer part of the diagnostic criteria. This issue of language development is however crucial for our purposes, since there is generally no history of language delay in HIP children (Vaivre-Douret, 2012). Thus, the clinical similarities mentioned above concern certain HIP children and children formerly diagnosed with AS rather than those diagnosed with HFA.

In summary, the aim of this article is to explore the literature in order to provide empirical supports for a better understanding of this clinical overlap between ASD and HIP, and then to propose a new line of investigation for further study.

## Methods

### Procedure

The procedures implemented for this literature review were as follows.

A computerized search was conducted using specific keywords processed by two databases: Pubmed/Medline and PsychInfo.

Reviews or meta-analyses were systematically searched and the most recent were selected. The original studies retained for this review came from peer-reviewed journals and were chosen according to: (1) the relevance of their content to the subject at hand (focus on abstracts, tables, discussions); (2) the methodology (participants, clinical assessments, materials used); (3) the publication date (recent studies were preferred); (4) the authors' contributions to a particular field (number of publications in one specific field, theoretical contribution). Studies without any standardized clinical assessment(s) for the diagnosis of ASD or HIP were excluded.

### Search strategy

An “autism lexical field” was defined as follows: “Autis^*^,” “ASD,” “Pervasive Developmental Disorders,” “High functioning Autism,” “Asperger Syndrome,” and a “giftedness lexical field”: Gift^*^,” “High ability,” “Intellectual high ability,” “High/Superior Intellectual Quotient,” “Intelligen^*^.”

In a first time, terms from the “autism lexical field” were associated with those from the “giftedness lexical field” in order to verify whether this subject of study, in any area of research, had been addressed before.

Then these two lexical fields were used in association with key words relating to the different subsections in the Results: “Cognit^*^,” “Psychometric^*^,” “Intellectual Quotient,” “Special skills/abilities,” “Talent,” “Mathematical/Verbal abilities,” “Attention,” “Sensor^*^,” “Emotion,” “Anxiety,” “Development^*^,” “Neurobiol^*^,” “Neurolog^*^,” “Cortical connectivity,” “Neuroanat^*^,” “Cytoarchitecture,” “Cerebral development,” “Foetal testosterone,” “Prenatal exposure,” “Hemispheric asymmetry,” “Neuropathology,” “Planum temporale,” “Superior Temporal Sulcus,” “Temporal Lobe,” etc.

These terms were also coupled with “High Functioning Autism” AND “Asperger Syndrome” in order to find articles in which a clear distinction between HFA and AS was made, enabling comparisons.

## Results

### Comparative data between children with HIP and/or ASD

To our knowledge, just one study has provided an empirical account of the differences between HIP children with and without ASD, providing some information concerning the differential diagnosis (Doobay et al., 2014). In this study, Doobay et al. recruited 81 HIP children (Verbal Comprehension and Perceptual Reasoning Indexes of the WISC-IV = or > 130). Forty received a diagnosis of ASD assessed by the Autism Diagnostic Interview-Revised (ADI-R) and the Autism Observation Schedule (ADOS). Mean ages were 10.76 years (*SD* = 3.26) for the ASD/HIP group and 9.43 years (*SD* = 2.30) for the non-ASD/HIP group. Parents, teachers, and participants completed the Behavioral Assessment System for Children 2nd edition (BASC-2). Parents and participants were also asked to complete the Vineland Adaptive Behavior Scales 2nd edition (Vineland-II). Concerning results from the Wechsler scales, no significant differences were found between the ASD group and the non-ASD group, except for Processing Speed Index (PSI), which was significantly lower in the ASD group (96.43 in ASD group vs. 110.41 in the non-ASD group). On the Vineland-II, the ASD group obtained significantly lower scores on the three subscales (“Socialization,” “Communication,” “Daily Living Skills”) in comparison with the non-ASD group who scored slightly above the average, and very much above on the “Communication” subscale. The greatest difference concerned the “Socialization” subscale for which scores were significantly below the average in the ASD group. The mean scores for the other two subscales were within the “Adequate” range (just below the average) in the ASD group. The BASC-2 is composed of several indexes, differing from one “version” (parent/teacher/participant) to another. Overall, the two groups exhibited significant differences on all but two subscales (“Anxiety” and “Conduct Problems”). The scores for the non-ASD group were generally in the average range, whereas the ASD group showed scores within the “At-risk" range (−1 to −2 *SD*) concerning the subscales “Depression,” “Attention problems,” “Hyperactivity,” “Adaptability,” “Activities of Daily Living” and “Social Skills”; and within the “Clinically Significant" range (−2 to −3 *SD*) for the “Atypicality” and “Withdrawal” subscales. These results highlight significant group differences between ASD/HIP children and non-ASD/HIP children. Despite high levels of ability, very high-functioning children with ASD showed difficulties in the adaptive and psychosocial domains, not found in non-ASD/HIP children. In the present study, the absence of an ASD/non-HIP group can be regretted because it would have shown whether these impairments were less pronounced among ASD/HIP children than among non-gifted ASD children.

In the above study, HIP children scored in the average range on the Vineland-II, but these results contrast with another study (Liratni and Pry, 2011), which highlighted adaptive difficulties among HIP children. In their original article, Liratni and Pry (2011), compared two groups of HIP children from a French sample (*n* = 35, mean age: 10 years 7 months): those who were consulting for socio-emotional and/or behavioral issues, and those who were not (control group of “healthy” HIP children). The psychometric profiles of the clinical group on the WISC-IV were more heterogeneous than those in the control group, and were characterized by considerable discrepancy between the VCI and the PRI (29.85 points) in favor of the VCI. The PSI was in the average range for both groups. The results on the Vineland scale showed that: (1) scores in the clinical group were significantly lower than those in the control group *and* below the average, except for the “Communication” subscale which coincided with the mean, the “Socialization” subscale was 1 *SD* below the mean and the “Daily Living Skills” subscale was 2 *SD* below the mean; (2) in the control group, “Communication” and “Socialization” were in the average range, but “Daily Living Skills” was 1 *SD* below the mean. This research demonstrates the existence of certain adaptive issues even among HIP children who were thought not to have any psychopathological disorders. A negative correlation was also found between PRI and the full scale score on the Vineland scale (Total Social Quotient) suggesting a link between good non-verbal abilities and social difficulties, which, according to the authors, to some extent recalls the HFA/AS profile, where they do not differentiate the two conditions.

This last study suggests that social interaction problems or general adaptive impairments may be associated with HIP, a result that is not found in Doobay's study in which it can be noted that the PSI score of the non-ASD/HIP group is about 10 points higher than the PSI in the two groups of HIP children in Liratni's study. Consequently, it can be supposed that the children with HIP in the first study had a more homogeneous IQ than the children in the second one. Conflicting data on the adaptive skills in HIP children could be also explained by a narrower interpretation of the diagnostic criteria for ASD in France/Europe, which in the French sample in Liratni's study might have led to the inclusion of children who, in other countries/circumstances, might receive a diagnosis of ASD in its least severe form.

Although these two studies cannot be directly compared, they show that ASD/HIP children encounter socio-adaptive difficulties not found in non-ASD/HIP children, and also that these difficulties may be present in some HIP children who have: (1) socio-emotional and/or behavioral issues and (2) a heterogeneous IQ profile. It can be regretted that it is impossible to know what proportion of children with ASD (if any) were included in this HIP group. However, ASD children were included, it can be supposed that these children would not present a typical form of autism, but precisely a “form” for which the diagnosis is open to discussion.

### Empirical support for clinical observations

#### Psychometric and cognitive features

The analysis of the psychometric characteristics of these populations is informative for the determination of different clinical features. The psychometric profile of children with HIP on the WISC-IV can be characterized by strong performances on the Verbal Comprehension Index (VCI) and by a Processing Speed Index (PSI) in the mean or lower range, with a trough on “Coding” (Kaufman, 1992; Bessou et al., 2005; Liratni and Pry, 2007, 2011, 2012; Lohman et al., 2008). These two features of the psychometric profile of children with HIP correspond to those presented by children with AS (Table 1). According to Huber (Huber, 2008), who makes no distinction between AS and HFA, this trough on “Coding” is also found in children with ASD who have a high IQ. In addition, these characteristics (very high VCI and PSI in the mean or lower range) appear to be constant over time, irrespective of the WISC version considered. However, low scores on the PSI may not always lead to the same clinical interpretation. Furthermore, inter and/or intra-scale IQ heterogeneity in the population with HIP tends to be the norm and not the exception (Jambaqué, 2004; Pereira-Fradin, 2004; Liratni and Pry, 2012). It is however possible that HIP subjects with homogeneous IQ profiles might be not taken into account because they are “doing fine” and thus are not often identified. Recent research (Guénolé et al., 2013a; Simoes-Loureiro et al., 2013) concludes that the more disharmonious is a profile, the greater the probabilities that the child will present neuropsychological or psychopathological disorders.

**Table 1:** **Index and subtest scores on the WISC-III in children with HFA and with AS (Ghaziuddin and Mountain-Kimchi, 2004; Koyama et al., 2007; Noterdaeme et al., 2010; Girardot et al., 2012; Planche and Lemonnier, 2012) and with HIP (Bessou et al., 2005)**.

	**Diagnosis Groups**		
	**HFA**	**AS**	**HIP**
Ghaziuddin and Mountain-Kimchi, 2004	*n* = 12	*n* = 22	–
Age (years; months)	12; 5	12; 3	
Full scale IQ *(SD)*	92.2 *(15)*	103.3 *(16)*	
Verbal IQ	91.15 *(13)*	107.4 *(12)*	
Information	8.7 *(3)*	13.0 *(3)*	
Similarities	10.0 *(3)*	12.0 *(2)*	
Arithmetic	6.3 *(3)*	9.9 *(3)*	
Vocabulary	9.2 *(2)*	11.6 *(3)*	
Comprehension	7.8 *(3)*	9.5 *(3)*	
Performance IQ	93.1 *(17)*	96.5 *(18)*	
Picture completion	9.0 *(2)*	11.3 *(3)*	
Picture arrangement	8.7 *(2)*	8.1 *(3)*	
Block design	10.5 *(5)*	11.0 *(4)*	
Coding	7.1 *(3)*	7.2 *(3)*	
Object assembly	9.2 *(2)*	10.5 *(4)*	
Girardot et al., 2012	*n* = 13	*n* = 18	–
Age (years; months)	11; 1	10; 8	
Full scale IQ *(SD)*	–	–	
Verbal IQ	66.5 *(11.1)*	114 *(8.5)*	
Information	4.9 *(3.2)*	13.0 *(2.7)*	
Similarities	6 *(2.5)*	13.0 *(2.3)*	
Arithmetic	–	–	
Vocabulary	5.5 *(3.2)*	13.10 *(2.15)*	
Comprehension	4.08 *(2.11)*	11.00 *(2.68)*	
Performance IQ	94.50 *(10.6)*	96.00 *(11)*	
Picture completion	9.42 *(2.43)*	11.08 *(2.63)*	
Picture arrangement	8.33 *(1.16)*	9.23 *(2.31)*	
Block design	10.90 *(2.67)*	10.06 *(3.10)*	
Coding	–	–	
Object Assembly	–	–	
Koyama et al., 2007	*n* = 37	*n* = 36	–
Age (years; months)	12; 7	12; 10	
Full scale IQ *(SD)*	94.6 *(13.5)*	98.3 *(14.1)*	
Verbal IQ	92.8 *(18.2)*	101.2 *(17.5)*	
Information	9.6 *(4)*	10.9 *(4.3)*	
Similarities	9.8 *(3.8)*	10.1 *(3.1)*	
Arithmetic	10.6 *(3.7)*	9.9 *(3.6)*	
Vocabulary	7.6 *(3.5)*	10.6 *(3.9)*	
Comprehension	6.2 *(2.9)*	9.2 *(3.4)*	
Performance IQ	97.9 *(15.1)*	95.4 *(14.5)*	
Picture completion	9.0 *(3.6)*	9.3 *(3.2)*	
Picture arrangement	8.5 *(3.1)*	9.3 *(3.3)*	
Block design	12.0 *(3.9)*	11.5 *(3.5)*	
Coding	9.2 *(4)*	7.5 *(2.7)*	
Object assembly	10.0 *(3.7)*	9.6 *(3)*	
Noterdaeme et al., 2010	*n* = 51	*n* = 55	–
Age (years; months)	10; 6	11; 2	
Full Scale IQ *(SD)*	94.0 *(9.6)*	104.1 *(14.3)*	
Verbal IQ	97.6 *(12.4)*	113.3 *(18.5)*	
Information	10.7 *(2.5)*	12.6 *(3.3)*	
Similarities	9.8 *(1.8)*	12.6 *(2.9)*	
Arithmetic	9.7 *(3.4)*	11.4 *(3.8)*	
Vocabulary	9.4 *(2.4)*	12.5 *(3.3)*	
Comprehension	7.3 *(2.4)*	9.6 *(3.4)*	
Performance IQ	92.8 *(11.6)*	96.5 *(16.2)*	
Picture completion	9.7 *(2.2)*	9.9 *(2.6)*	
Picture arrangement	6.6 *(2.2)*	8.6 *(3)*	
Block design	9.9 *(2.6)*	11.9 *(1.9)*	
Coding	7.5 *(2.2)*	8.1 *(2.8)*	
Object assembly	8.8 *(2.8)*	8.9 *(3.3)*	
Planche and Lemonnier, 2012	*n* = 15	*n* = 15	–
Age (years; months)	8; 6	8; 3	
Full Scale IQ *(SD)*	98.07 *(16.28)*	105.53 *(16.87)*	
Verbal IQ	89.13 *(17.56)*	112.33 *(17.10)*	
Information	8.40 *(3.70)*	13.27 *(2.66)*	
Similarities	9.27 *(3.58)*	13.13 *(3.66)*	
Arithmetic	6.07 *(2.60)*	9.27 *(2.74)*	
Vocabulary	9.67 *(3.04)*	11.87 *(3.18)*	
Comprehension	7.53 *(4.39)*	12.13 *(3.50)*	
Performance IQ	109.07 *(13.52)*	96.53 *(14.74)*	
Picture completion	13.40 *(1.99)*	13.47 *(3.14)*	
Picture arrangement	11.60 *(3.91)*	10.13 *(3.11)*	
Block design	12.47 *(2.39)*	9.53 *(2.90)*	
Coding	7.00 *(3.85)*	4.00 *(2.80)*	
Object assembly	11.93 *(2.94)*	10.33 *(3.36)*	
Bessou et al., 2005	–	–	*n* = 245
Age (years; months)			8; 5
Full scale IQ *(SD)*			138.37 *(–)*
Verbal IQ			137.84 *(–)*
Information			15.23 *(–)*
Similarities			17.02 *(–)*
Arithmetic			13.84 *(–)*
Vocabulary			15.81 *(–)*
Comprehension			16.84 *(–)*
Performance IQ			126.00 *(–)*
Picture completion			14.79 *(–)*
Picture arrangement			14.11 *(–)*
Block design			13.95 *(–)*
Coding			11.14 *(–)*
Object assembly			13.57 *(–)*

HFA, High Functioning Autism; AS, Asperger Syndrome; HIP, High Intellectual Potential; SD, Standard Deviation; IQ, Intellectual Quotient; “–“, No or not available data. Italic values correspond to Standard Deviations (SD).

A large proportion of heterogeneous profiles was also evidenced by Liratni and Pry (2012). The authors recruited 60 children with HIP (mean age: 9 years 8 months), on the basis of the WISC-IV, from different school backgrounds. The results showed, for a mean FSIQ of 135.1: a mean VCI of 140.6, a mean PRI of 120.9, a mean Working Memory Index (WMI) of 121.7, and a mean PSI of 113.4. Eighty-seven percent of the children in this sample presented a difference of more than 23 points between the highest and the lowest index (an average of 35.6 points over this sample), making their FSIQ impossible to interpret (Flanagan and Kaufman, 2004). Five children presented statistically very rare differences ranging from 60 to 69 points. A correlation matrix highlighted a negative relationship between “Vocabulary” and “Coding.”

In the field of ASD, it is important here to dissociate HFA and AS. Several studies published before the issue of DSM-5 showed the existence of significant differences between psychometric profiles of children with HFA and those with AS: on the Wechsler Intelligence Scale for Children 3rd edition (WISC-III) (Wechsler, 1991), children with HFA exhibited better visual-spatial abilities (Performance Intellectual Quotient or PIQ) than verbal skills (Verbal Intellectual Quotient or VIQ) and the reverse was observed in children with AS (Ghaziuddin and Mountain-Kimchi, 2004; Koyama et al., 2007; Noterdaeme et al., 2010; Girardot et al., 2012; Planche and Lemonnier, 2012; Chiang et al., 2014). Their scores on the different indexes and subtests are detailed hereafter (Table 1).

Table 1 shows on the one hand the scores of AS and HFA children on the WISC-III drawn from five different studies comparing these two populations (Ghaziuddin and Mountain-Kimchi, 2004; Koyama et al., 2007; Noterdaeme et al., 2010; Girardot et al., 2012; Planche and Lemonnier, 2012), and on the other the scores of HIP children derived from one study (Bessou et al., 2005).

The results show that Full Scale IQ is higher in AS children than in HFA children in 4 of the 5 studies. The Verbal IQ score is also higher than the Performance IQ score in AS children in all these studies, and Performance IQ is higher than Verbal IQ in HFA children in 4 of the 5 studies. In addition, according to these results, Verbal IQ is always higher in children with AS than in those with HFA. In contrast, Performance IQ is higher in children with HFA than in those with AS in only 2 of the 5 studies. According to these studies, in children with AS, the subtest that is the most often well-performed is “Information” from the verbal scale, and the most often failed is “Coding.” In the children with HFA, “Block Design” from the performance scale constitutes the best performed subtest in the most of these studies, whereas “Coding” appears to be the most often failed.

As expected, the scores obtained by children with HIP in the study by Bessou et al. are globally higher than those of children with AS or HFA. In this HIP group, Verbal IQ is higher than Performance IQ in 81.7% of cases, and “Similarities” obtained the highest score whereas “Coding” obtained the lowest.

It can be observed that Verbal IQ reflects strength of the cognitive profile among children with AS and those with HIP, but not among children with HFA. “Coding” appears to be the least well-performed subtest in the HFA, AS and HIP groups.

Marked heterogeneity can be noted across the subtests in the three groups: (1) In the HFA group, the inter-subtest difference (highest score–lowest score) ranges from 4.1 points to 6.82 points according to the studies (mean 5.46); (2) In the AS group, the inter-subtest difference ranges from 3.87 points to 9.47 points (mean 5.53); (3) In the HIP group, the difference is 5.88 points. A difference of more than 5 points is regarded as significant at *p* = 0.05 (Wechsler, 1991).

This inter-subtest heterogeneity appears to be a psychometric feature common to ASD and HIP. These results, however, should be considered with caution, in particular because they are based on a small number of studies and also because there is no specific psychometric profile associated with a particular population.

#### Specific abilities or “special skills”

This inter-subtest heterogeneity is related to the emergence of “Special Skills” which concern both HIP and ASD subjects. In a study by Howlin et al. (2009), out of 125 individuals with ASD in their sample (mean age: 24.1 years, *SD* = 8.6), 28.5% presented out-of-the-ordinary skills, 17% presented out-of-the-ordinary cognitive abilities (score > + 2 *SD* on at least one subtest in the WISC-IV), and 11.6% presented both. The results also show that no subject who obtained a non-verbal IQ below 50 presented special skills, and the diagnostic criterion “restricted, repetitive and stereotyped patterns of behaviors, interests and activities” bore no particular relationship to special skills or special cognitive aptitudes in this study. This suggests that at least the 2nd and 3rd groups presented inter-subtest heterogeneity on the WISC-IV. Another study (Meilleur et al., 2015) suggests that outstanding abilities, which are referred to in the ADI-R as “Special Isolated Skills” (SIS), are a recurrent feature of ASD. In this study, the presence of SIS was evaluated via questions 88–93 in the ADI-R. The results show that, in the sample of individuals with ASD (*n* = 254, mean age: 20.81 years), the prevalence of at least one SIS was 62.5%. Among these, 71.7% had more than one SIS. The results also highlight an increase in SIS prevalence with intelligence level and age.

In the study by Baron-Cohen et al. (2009), the first characteristic predisposing to the development of a talent is “hyper-systemizing,” which, in association with poor central coherence, leads to devoting extra attention to detail. According to the authors, attention to detail in ASD arises from sensory hypersensitivity, in particular visual, and from the preferential use of an analytical processing mode. Ruthsatz and Urbach (2012) showed that in their sample of talented subjects with HIP (mean age: 13.63 years), half had a member of their family (direct, 1st or 2nd degree) who had received an ASD diagnosis. These participants scored on average significantly higher than the control group on the Autism Quotient questionnaire (AQ) (Baron-Cohen et al., 2001), but lower than the ASD group, except for the sub-category “attention to detail” which was higher than in the ASD group. Results from a second study (Ruthsatz et al., 2015) point to genetic evidence suggesting a shared etiology between ASD and “Prodigies.” According to this research, a locus on chromosome 1 could be related to the emergence of both autism and “prodigies” in a same family. These results are consistent with the high prevalence of ASD children found among the HIP group in the Doobay's study (Doobay et al., 2014).

In addition, according to one study (Yun et al., 2011), teenagers with high abilities in mathematics had difficulties in sharing their centers of interest and in interacting with their peers. The results of this study showed that strategic but socially inappropriate behaviors (aiming to win the game) in the HIP group reflected their difficulties in decision-making processes in a social context.

#### Attention

Another common clinical feature concerns attentional processes. Some children with HIP and those with ASD are described by having “fluctuating attention,” in other words they can stay focused for hours on their interests but show great difficulty staying concentrated on certain tasks. They tend also to be preoccupied by internal thoughts. The question of the co-occurrence of Attention Deficit/Hyperactivity Disorders (ADHD) and ASD, or ADHD and HIP, has often been addressed (Antshel et al., 2007; Loureiro et al., 2009; Grzadzinski et al., 2011; Martin et al., 2014; Ronald et al., 2014; Whitaker et al., 2015).

According to Martin et al. (2014), there is a significant overlap of biological processes in ADHD and ASD. Grzadzinski et al. (2011) identified a subgroup of children with ADHD among children with ASD. The presence of ADHD in children with HIP is still controversial, but according to some studies this co-occurrence is not anecdotal. Antshel et al. (2007) showed that ADHD was a valid diagnosis in presence of a high IQ, since these children present psychiatric and behavioral features consistent with the diagnostic criteria of ADHD in average IQ children. In addition, Loureiro et al. (2009) demonstrated that HIP children with ADHD had a particular neuropsychological profile. In this study, 3 groups of HIP children (*n* = 45), aged between 7 and 11 years, were compared: (1) HIP children with a homogeneous profile on the WISC-III (less than 12 points between VIQ and PIQ); (2) HIP children with a heterogeneous profile (VIQ > PIQ); (3) HIP children with a very markedly heterogeneous profile (more than 20 points). The first group obtained better scores on attention tasks than the second and third groups. On the “Digit Span” subtest, the first group performed better than the 2nd and 3rd groups, but only the first and third groups significantly differed from each other. In addition, the diagnostic criteria for ADHD (poor working memory, failure on at least 3 attention tasks, Diag-80, etc.) were examined across the whole sample. The results show that among the 45 children with HIP, 13 met the criteria for the diagnosis of ADHD: 4 belonged to the 2nd group and 9 to the 3rd group. The authors highlight a relationship between heterogeneous IQ and disharmonious neuropsychological profile.

Different features of ADHD seem to be regularly associated with ASD symptomatology but also with HIP, especially when the IQ profile is heterogeneous. Attention deficits, or at least atypical attentional processes, could emerge as another neuropsychological commonality between some ASD children and some HIP children. In addition, a heterogeneous IQ profile could constitute a first indicator of possible associated disorders in HIP children.

#### Sensory modulation

Sensory modulation specificities are also mentioned in the introduction as being common to ASD and some HIP children. Numerous studies examined the unusual sensory modulation in ASD which appears to be a part of the symptomology since the DSM-5 integrated it in criterion B. According to Leekam et al. (2007), using the Sensory Profiles (Dunn, 1999), in their sample, 94% of subjects with ASD (Low Functioning Autism group: *n* = 16, mean age 86.16 months; High Functioning Autism group: *n* = 17, mean age 87.00 months) showed atypical sensory processing in numerous conditions. These atypical perceptions and reactions range from a lack of responsiveness (hyposensitivity) to sensory hyper-reactivity (hyperesthesia) which play a role in the preference for a certain type of stimulation and can result in idiosyncratic behavior. This atypical sensory processing persists over time and is not dependant on IQ level (Green et al., 2013; Tavassoli et al., 2014).

In the field of HIP, this criterion is practically absent from the literature, whereas it is often cited by parents and clinicians. One study (Gere et al., 2009) highlighted specific sensory profiles in children with HIP (*n* = 80; mean age: 8.7 years) using Dunn's questionnaire. The authors performed mean comparisons (*t*-test) between the HIP group and the norms. On the 14 sections of the Sensory Profile, the scores of the children with HIP differed significantly from the norm in all sections except “Visual Processing” and “Threshold for Response.” According to the authors, these results show a greater sensitivity to sensory stimulations, resulting in significantly more intense emotional reactions in subjects with HIP than those observed in typical children. The authors also concluded that children with HIP sometimes present sensory information processing disorders that could cause functional problems. Other researchers (Miller et al., 2007) attribute sensory hyper-responsiveness to behavioral parameters such as impulsiveness, aggressiveness, withdrawal, or avoidance of stimulations, which are classic symptoms in ASD.

#### Emotional regulation and adjustment

Explosive emotional reactions are a clinical sign of ASD. Indeed, several studies have concluded that emotional regulation impairments are a key feature of ASD (Rogers et al., 2007; Rieffe et al., 2011; Samson et al., 2012, 2015a,b). Rogers et al. (2007) administered the Interpersonal Reactivity Index (IRI) (Davis, 1980), a 28-item self-report questionnaire that measures both cognitive and affective empathy, to 21 individuals with AS (mean age: 42.9 years, *SD* = 10.6) and 21 controls (mean age: 41.9 years, *SD* = 13.8). The results show that AS individuals scored significantly higher on “Personal Distress” (IRI affective scale) than the control group, and indicate that AS individuals are not impaired in affective empathy but tend to become anxious faced with emotional responses from others. Individuals with AS lack strategies, such as cognitive reappraisal, to reduce the negative effects of someone else's emotions. Consequently, they shift toward egocentric feelings and thoughts in order to reduce or escape from the aversive stimulation. They also experience more negative affects such as anger or fear. These findings and conclusions were relayed in several studies (Rieffe et al., 2011; Samson et al., 2012, 2015a,b).

Overall, studies on the socio-emotional competences of HIP children have not reached any consensus, and two antagonistic views are upheld in the literature. According to the first, HIP children, on account of their emotional immaturity and their difficulty in managing their emotions, lack the aptitude to establish social relationships that are stable over time. The second view is that HIP children exhibit competences in the socio-emotional domain that are at least in the norm (Lautrey, 2003). From a clinical viewpoint, children with HIP are often described as “hyper-sensitive,” which supports the first assumption. Indeed, they tend to overreact when faced with things that are in appearance anecdotal, and they also may exhibit explosive anger or feel deep distress, etc. Emotional regulation processing has nevertheless not been empirically explored in HIP. Some studies exploring emotional characteristics in children with HIP have reached conflicting conclusions as mentioned above (Guignard and Zenasni, 2004; Guignard et al., 2012; Guénolé et al., 2013a,b).

Anxiety traits were analyzed in 111 clinically referred HIP children (mean age: 9.6 years, *SD* = 1.3) using the French version of the Revised-Children's Manifest Anxiety Scale (R-CMAS) (Guénolé et al., 2013b). The results showed no significant differences between HIP children and the norms. The same conclusions were reached in another study (Guignard et al., 2012). Guénolé et al. (2013a) administered the Child Behavior Checklist (CBCL) to a clinically-referred population of 143 children with HIP (mean age: 9.3, *SD* = 1.00). A comparison of means showed significantly higher scores on all the subscales of the CBCL in the HIP group, including the “Anxious/Depressed” and “Social problems” sub-categories. The authors also compared HIP children with a “Significant Verbal-Performance Discrepancy” (SVPD) with non-SVPD children. The results show significantly higher scores on the “Externalized Problems” scale in the SVPD group, which is congruent with previous conclusions (Loureiro et al., 2009). The authors concluded that a significant IQ discrepancy reflected a heterogeneous developmental pattern associated with an increased risk of behavioral and emotional problems. They added that SVPD is a feature of Asperger Syndrome (even when not “specific”) that shares characteristics with some children with HIP like: “*verbal precocity, hyperlexia, […], absorbing interests in specialized topics (with limited social sharing), social withdrawal, anxiety, excessive perfectionism, perceptive hypersensitivity, and motor clumsiness. Intellectual giftedness is common in mild forms of [Pervasive Developmental Disorder] PDDs.[…] As PDDs are thought to represent the high-level co-occurrence of continuously distributed quantitative traits, it could be hypothesized that a significant proportion of clinically referred gifted children may be situated at the border of such developmental atypicalities.”* The authors of this study underline that clinically-referred HIP children form a group with moderately pathological behavior and point out their “*nosological orphan”* status.

In the DSM-IV-TR, Autistic Disorders, including HFA, and Asperger Syndrome, were referred to as “Pervasive *Developmental* Disorders.” Several studies cited in this review show that some children identified with HIP are clinically-referred and seem to fit a particular profile characterized by at least IQ discrepancies, problems in fixing attention, atypical sensory modulation, and difficulties in emotional regulation. As in ASD, these symptoms appear to be related to a disharmonious pattern of development.

## Developmental characteristics of children with HIP

Greater maturity of neuro-sensory-motor, cognitive and language functions was observed in children between 0 and 36 months of age subsequently identified as HIP (Vaivre-Douret, 2004a,b, 2011, 2012; Loureiro et al., 2010). On a strictly neuro-motor level, Vaivre-Douret demonstrated that from birth, infants subsequently identified as HIP with a homogeneous WISC-profile showed active mobile exploration requiring efficient oculomotor characteristics, as well as efficient oculocephalogyric pursuit, suggesting early axial neuro-motor maturation (Vaivre-Douret and Jambaqué, 2006). Between 0 and 2 years of age, an early disappearance of primitive reflexes was observed, as well as an advance in axial cephalocaudal and proximo-distal maturation (Vaivre-Douret, 2004b). Developmental data confronted with Brunet-Lézine's scales for normal child development also shows early acquisition of primary motor skills—various motor skills were acquired on average 1 to 2 months (+1 *SD* to +2 *SD*) earlier in the children that later exhibited HIP (Vaivre-Douret, 2004a). Furthermore, a large number of difficult pregnancies, and a large proportion of prematurely born infants are reported (18.6% of HIP children in one sample vs. 5.9% among typical children), including a large proportion of infants with a weight, height and cranial circumference that correspond to a percentile range equal to or greater than 90 (with respect to gestational age), in both new-born girls and boys (Vaivre-Douret et al., 2010). HIP development could be linked to prenatal exposure to certain hormones (Mrazik and Dombrowski, 2010), which also appear to play an important role in the etiology of autistic disorders.

Planche and Gicquel (2000) established that, at the “formal operational stage” according to Piaget, performance discrepancies existed in HIP children on intra-individual level. Globally, once a developmental threshold is crossed, HIP children master operations related to that stage more quickly without necessarily being able to access the next stage much faster. In the period following access to a new stage, the different notional domains develop simultaneously at varying paces, causing a certain disharmony, and then become gradually homogenized. Access to a new developmental stage is reached by acquisitions in a particular notional field acting as a trigger. Likewise, HIP children are thought to be able to access the “concrete operational stage” by a different pathway from that of typically developing children, that is to say by mastering the invariance principle, which they acquire and generalize earlier than their peers of the same age, at a time when the other logical concepts are not yet present (Planche, 2008). In accordance with this model, different developmental pathways need to be considered for different types of children.

**Figure 1:**
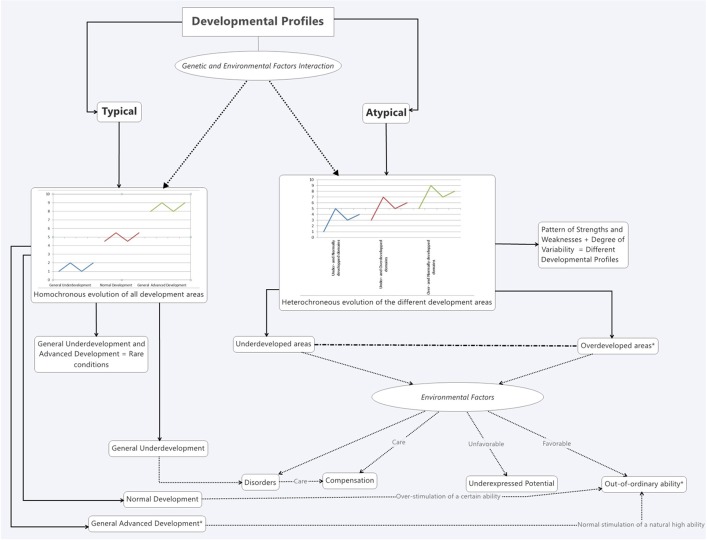
**The typical and atypical general development profiles hypothesis**.

This suggests that the development of HIP children appears to be on the one hand “accelerated,” in case of homogeneous IQ profiles, according to Vaivre-Douret, but on the other hand should be considered in terms of unusualness, characterized more by irregularities and asynchronies, than by advanced development. These findings support the idea that some children identified with HIP may present moderate “*Developmental Disorders”* (See below Figure 1). These overlaps highlighted so far seem to be present at more fundamental levels. Indeed, work on cerebral development and studies conducted in the field of neurobiology provide interesting data concerning the etiology of ASD and HIP.

## Neurodevelopment and neurobiological aspects of ASD and HIP: toward a common etiology?

Our starting point is the Geschwind-Behan-Galaburda Model (GBG Model) (Geschwind and Behan, 1982; Geschwind and Galaburda, 1985). A first study by Geschwind and Behan (1982) established that a frequent association between left-handedness, auto-immune diseases and learning disorders in male subjects was attributable to fetal testosterone. According to this model, fetal testosterone slows down the maturation of the left hemisphere. Exposure to abnormally high levels of testosterone induces abnormalities in the left temporal lobe (in particular Wernicke's area and the Planum Temporale). In addition, fetal testosterone has a repressive effect on the thymus and impacts the maturation of the immune system. In this study, the authors mention celiac disease (an auto-immune disease) as being recurrent in autistic children, along with other auto-immune diseases for which the prevalence is higher in this population (Sweeten et al., 2003).

The GBG model has been enriched by a large number of studies (Winner, 2000), particularly one by Benbow (1986) who noted a large proportion of left-handed or ambidextrous individuals who were asthmatic, allergic or myopic in an HIP population, linking these physiological characteristics to high exposure to fetal testosterone. This larger proportion of left-handed individuals among High IQ subjects appears in the conclusions of other studies (Annett and Kilshaw, 1982; Geschwind and Behan, 1982; Lewandowski and Kohlbrenner, 1985; Casey et al., 1992) and is also found in ASD populations (Hauck and Dewey, 2001; Lindell and Hudry, 2013; Preslar et al., 2014). Using a dichotic listening task, O'Boyle and Benbow (1990) demonstrated that typical children tended to identify syllables better with their right ear, processed by the left hemisphere, while HIP children recognized the syllables with just as much accuracy in the left ear, processed by the right hemisphere, as in the right. An EEG study (O'Boyle et al., 1991) confirmed this neuro-functional characteristic of HIP children, consisting in a greater involvement of the right hemisphere, and demonstrating a degree of hemispheric equipotentiality or lack of asymmetry. Further investigations (O'Boyle et al., 1995; Singh and O'Boyle, 2004; O'Boyle, 2005, 2008) highlighted not only a greater involvement of the right hemisphere in HIP subjects compared to typical subjects, but also a more efficient hemispheric interconnectivity than average, supposedly supported by anatomical and functional differences of the corpus callosum.

Furthermore, other studies also highlighted less marked hemispheric asymmetry in ASD subjects, which could be attributed, according to a MEG study, to structural aberrations in the left Planum Temporale (Wilson et al., 2007) already observed in previous studies on autism. In fact, studies on both children and adults have revealed that unlike what is observed in typical subjects, the left and right Planum Temporale in ASD subjects are equal in size (Rojas et al., 2002, 2005). It is interesting to note that similar results were obtained with schizophrenic patients: a greater prevalence of left-handed or ambidextrous subjects and hemispheric equipotentiality, highlighted by a dichotic listening task, and lesser Planum Temporale asymmetry (Sommer et al., 2001), the same also being observed with dyslexic children (Bloom et al., 2013).

Baron-Cohen and their collaborators consider that the cerebral developmental pathway among ASD subjects corresponds to an extremely masculinised brain, theorized in the model of the “extreme male brain theory” (EMB theory) (Baron-Cohen et al., 2005, 2011; Auyeung et al., 2013). Testosterone modifies neuronal connectivity, acting directly on DNA. It increases the formation of dendritic spines via a process mediated by BDNF (brain-derived neurotrophic factor). Androgen receptors are present at the start of the third trimester of pregnancy and their expression is very high, especially in the temporal lobe (Baron-Cohen et al., 2005) which we know to be implicated in language and social stimulus processing, and which presents functional abnormalities in ASD subjects (Saitovitch et al., 2012; Alaerts et al., 2014). Furthermore, an endocrinology study (Palomba et al., 2012) showed that the probability of giving birth to a child developing ASD is higher in women presenting hyperandrogenism with polycystic ovary syndrome.

The increased growth of dendritic spines is coherent with other research on cyto-architecture and neuronal connectivity in ASD. A certain number of studies demonstrated that atypical neuronal connectivity in ASD is characterized by under-connectivity in long-range circuits (the longitudinal fasciculi in particular) and by over-connectivity in local microcircuits, acquiring new functional properties but affecting complex information integration (Just et al., 2007; Casanova and Trippe, 2009; Schipul et al., 2011; Maximo et al., 2014). A very recent study (Tang et al., 2014), in a post-mortem analysis of ASD subjects' brains, highlighted increased dendritic spine density combined with reduced spine pruning in layer V pyramidal neurons within the Broadmann Area 21 (temporal cortex), a region involved in social processing.

These results are congruent with those of Markram and Markram (2010) who, on the basis of their previous research, proposed a new neurobiological model of autism (“The Intense World Theory”). From an animal model of autism generated by intra-peritoneal injection of valproic acid (VPA) in pregnant rats, the authors observed neuronal over-connectivity in microcircuits in different regions of the neocortex (in particular the prefrontal and somatosensory cortex) and in the amygdala, among the offspring exposed to VPA. This over-connectivity is combined with hyper-reactivity and hyper-plasticity of these circuits, in turn sustained by glutamatergic system alterations because of over-expression of the NMDA receptor subunits NR2A and NRB2, implicated in memory and learning. According to the authors, these characteristics have consequences in the cognitive and behavioral areas and explain in particular the hyper-attention to detail, withdrawal into a secure environment characterized by the immutability resulting from adverse sensorial stimuli, emotional hypersensitivity and anxiety. This notion of hyper-reactivity at the core of the Intense World Theory recalls Dabrowski's “over-excitability” (1964) later returned to by Ackerman (Ackerman and Moyle, 2009) for its relevance in identifying HIP children. In fact Dabrowski noted a strong propensity in these subjects to entertain an intense rapport with the world and to manifest *over-excitability* in response to environmental stimulations, which is in line with the conclusions of Gere.

Finally, we can mention the neurodevelopmental hypothesis set out by Mrazik and Dombrowski (2010), for whom HIP originates from an atypical cerebral organization for which the etiology, along the same lines as ASD, learning disorders or even schizophrenia, could result from prenatal exposure to different types of events and molecules (influenza virus, known for is implication in schizophrenia, testosterone, fever, etc.) between the 2nd and 3rd trimesters of pregnancy. These prenatal exposures influence neuronal migration, proliferation, differentiation, myelinisation, and apoptosis, enabling the development of certain cerebral areas at the expense of others, and generating cortical symmetry, left hemisphere volume reduction, right hemisphere enhancement and a thickening of the corpus callosum. The authors suggest a biologically plausible hypothesis according to which the same neurobiological factors could contribute to the development of both neuro-psycho-pathologies and HIP, where current debate focuses on the hypothesis of a more favorable terrain for the emergence of these disorders.

## Discussion

This article first of all underlines a near total absence of data on the similarities between ASD and HIP, despite growing clinical interest and the consequent need for empirical assessment. Obviously, this review does not enable direct comparison between ASD and HIP because the studies cited are not designed for that purpose, but it highlights several commonalities across numerous research areas (behavioral/clinical domain → neurobiological domain) which encourage further pursuit of this question.

This review of the literature based on a cross-sectional approach provides a more coherent overview of clinical observations and the results of empirical studies which are summarized hereafter (Table 2). It first of all appears that children with ASD and a large proportion of children with HIP present heterogeneous IQ performances on the Wechsler scales. Although this characteristic is not specific to the two populations, it reflects cognitive strengths and weaknesses. This discrepancy could be found in other domains of ability and it could explain the presence of “Special Isolated Skills” (SIS). The prevalence of SIS among subjects with autism is, according to numerous studies, greater than in other clinical populations. Concerning SIS, no research has been conducted on HIP children, who by definition possess abilities above the norm, at least in certain cognitive domains. Yet it would be valuable to gain knowledge on the underlying mechanisms for the development of SIS or particular talents from the parallel study of ASD and HIP populations.

**Table 2:** **Summary of similarities and differences outlined by the literature between HFA and AS, and between AS and HIP**.

	**HFA/AS**	**AS/HIP**
Similarities	- “Coding” (WISC) < Norm	-Verbal Skills/Extended Vocabulary
	-Heterogeneous WISC profile	-Heterogeneous WISC profile
	-Special isolated Skills	-Attention problems
	-Attention problems	-Atypicalities in sensory modulation
	-Atypicalities in sensory modulation	-Emotion regulation impairments
	-Emotion regulation impairments	-High prevalence of lefthanders
	-High prevalence of lefthanders	-Greater involment of the RH
	-Greater involment of the RH	-Dysharmonious Developmental trajectory
	-Dysharmonious Developmental trajectory	
Differences	-FSIQ (AS > HFA)	-FSIQ (HIP > AS)
	-Verbal Skills (AS > HFA)	-Socio-adaptative Skills (HIP > AS)
	-Severity level of the autistic symptomatology (HFA > AS)	-Severity level of the autistic symptomatology (AS > HIP)

HFA, High Functioning Autism; AS, Asperger Syndrome; HIP, High Intellectual Potential; RH, Right Hemisphere; FSIQ, Full Scale Intelligence Quotient.

This review of the literature has also enabled alternative conceptions of “empathy” in autism to be approached. According to numerous recent studies, people with autism could have deficit in terms of “cognitive empathy,” but not in terms of “affective empathy.” The difficulty managing affects generated by emotional experiences in others could lead to personal distress reactions, characterized by withdrawing from the aversive stimulation. A flight reaction or an intense emotional reaction both reflect the difficulty in differentiating one's own emotional experience with that of another. Emotional hyper-reactivity has been more often described among HIP children, viewed as being “edgy,” and this could be explained by sensory hyperaesthesia. Indeed, the amygdalae, one of the functions of which is the detection of the emotional valence of a stimulus, or more specifically the basal-lateral nucleus, receive numerous afferences from the thalamus, a relay structure for all sensory information. This is why the amygdalae are described as the “gateway to the sensory processing of emotions.” The sensory atypicalities in autism, now well-documented, today appear as diagnostic criteria in the DSM-5, and could offer the means whereby emotional processes could be studied in this population, as well as in the HIP population, which probably has common characteristics in the sensory domain, and possibly in the emotional domain. However, research on sensory modulation in HIP is as yet very inadequate.

The same applies for emotional processes, where exploration has produced a fundamental contradiction: there seem to be HIP children who are socially well-adapted and present competences that are at least in the norm in this area, and conversely HIP children presenting socio-emotional difficulties where the etiology is not yet clear. It can be noted that from a clinical viewpoint, HIP children who encounter socio-emotional difficulties very often present other clinical features, such as the presence of specific interests of a non-social type, tendency to withdrawal into an often “rich interior world,” considered to be secure by the child, all of which recalls autistic symptoms. On account of this tendency, ASD and/or HIP children are often considered to have attention problems. In certain cases, an additional diagnosis of ADHD is made. Numerous studies do show that the prevalence of ADHD is high among ASD subjects, and there is disagreement on the results for HIP subjects.

This review of the literature has enabled a certain profile of children to be sketched out, and a confrontation of clinical observations with empirical data. Nevertheless, the parallels are not altogether clear, on account of certain limitations linked to the present context, and in particular the conceptual blur concerning the definition of HIP.

### Limitations and perspectives

That data in the area of HIP is as yet inadequate, and this is the first major limitation of this review. The lack of interest in the subject in research can perhaps be explained by the fact that for a long time the term “giftedness was mainly related to the field of education, and aroused interest mainly among parents, teachers and a few clinicians. In fact, the theme was for a long period considered too “unscientific” to be broached. However, the neuropsychological, socio-emotional and sometimes psychomotor difficulties encountered in these children, ever more numerous in consultation centers, brought the issue into the field of psychopathology and developmental disorders. Even so, the theme is still new, overlaps other fields of research, and is the subject of disagreements. These disagreements concern in particular the question of whether HIP is related to one or several clinical entities, remaining to be defined, or whether it is a cross-sectional phenomenon appearing conjointly in various known pathologies, or independently from them, i.e., among “healthy” subjects. In this review, the few studies available on HIP necessarily led to the consideration of certain publications in which the methodological quality did not equal that of the studies on autism. Indeed, the large number of studies on autism enables more stringent selection criteria, such as the systematic presence of a control group.

Another limitation in the present review is the conceptual blur around the term “giftedness.” The conceptions of “giftedness” mentioned in the introduction are related to different phenomena and it currently seems crucial to clearly distinguish between them. Because of this, only research concerning individuals with an IQ of 130 or more were retained here. There are even so conflicting results across studies supposedly studying the same population. The reason for this is certainly that the definition of HIP based on a FSIQ score of 130 or more remains problematic. Indeed, in practice, most children with HIP consulting in a clinical structure present a heterogeneous profile and thus a statistically non-significant FSIQ. These children are identified by most of clinicians as “HIP” or as “gifted,” but do not correspond strictly to the theoretical definition. Consequently, identifications are arbitrary: can we talk about HIP when just one Index is above 130; or when the VCI and the PRI are above 130, regardless of the other indexes? Do we consider that a FSIQ score should be calculated despite marked discrepancies between indexes? And if not, where do we place the threshold? At 15 points according to Wechsler (2003)? At 23 points according to Flanagan and Kaufman (Flanagan and Kaufman, 2004)? Currently, HIP is liable to be identified in all these cases, according to the clinician's positioning. These clinical issues have a considerable impact on research, in particular because the lack of consensus on the semantic level has consequences on inclusion criteria. This point does indeed constitute another limitation of this review, since it then becomes difficult to know precisely what types of children make up the HIP groups in the studies cited.

Certain studies did however attempt to compensate for this conceptual blur by separating “clinically referred” HIP children from HIP children presenting no associated disorders. Although inadequate, this procedure nevertheless enabled socio-adaptive difficulties to be evidenced in certain children, leading to questionings on the nature of these difficulties also occurring in ASD children. Among the studies presented in this article, some were based upon another assumption and examined homogeneous and heterogeneous IQ profiles separately, and they showed that they could constitute differentiated neuropsychological and developmental profiles.

Heterogeneous IQ is a feature shared by ASD children and by a large proportion of HIP children, and it seems to be related to associated disorders, in particular developmental disorders. Indeed, this literature review, as a result of its cross-sectional approach, suggests that the clinical similarities observed between certain HIP children and certain ASD children stem from a more fundamental common base, and in particular certain characteristics of brain development determining the overall development of the individual. As in ASD, which is classified as a developmental disorder, certain HIP children have a developmental trajectory characterized by a degree of disharmony in the progression of different categories of acquisitions. From a genetic viewpoint, Ruthsatz et al. (2015) have gone as far as suggesting a common etiology between ASD and “child prodigies” in their recent study.

In summary, this cross-sectional approach affords an overview of the issue and raises two important considerations: (1) HIP could cover different developmental profiles. One of them seems to be more likely to develop associated disorders; (2) Clinical observations, which initially pointed to an overlap between ASD and HIP, seem to be tending toward a more fundamental relationship between ASD and a certain form of HIP.

### Suggestions for further lines of research

It is possible to consider this question of the similarities between ASD and HIP from a more holistic point of view and to integrate it into a “General Hypothesis” that also affords a differentiation between different profiles of “HIP.” Indeed, two large categories of general development profiles could be identified (Figure 1), one “typical” and one “atypical.” According to this hypothesis, the first category could be characterized by a harmonious and synchronous evolution of all areas of development (including cognition, socio-emotional development, psycho-motor development, etc.) with an overall progression rate that varies for each individual. These progression rates would then determine the individual's global level of ability which ranges from “general under-development” to “general advanced development.” The second category could be characterized by heterochrony in the evolution of the different areas of development. This heterochrony involves an independent progression rate in the different areas of development. Some of them may match the norm, whereas others may be retarded or over-developed. They would thus form a pattern of strengths and weaknesses reflecting different atypical developmental profiles, among them ASD, “Learning Disorders,” ADHD, etc. These two large categories of profiles could be evidenced by a complete developmental assessment examining each function in each area of development (e.g., cognitive, socio-emotional, psycho-motor, etc.).

Concerning the different HIP profiles, again according to this hypothesis, the asterisks (^*^) would each correspond to a particular definition of “giftedness”: (1) Generally high aptitudes and harmonious developmental evolution (homogeneous high abilities on complete development evaluation including IQ); (2) Development of a particular talent as the result of the stimulation of one or more ability. In this case, the stimulation intensity would depend on “how far” the ability is *naturally* developed (cf. Differentiated Model of Giftedness and Talent, Gagné, 2004); and (3) Atypical neurodevelopmental profile characterized by some very marked strengths and by other normal or below-average abilities. The first profile is thought to be a fairly rare condition (about 2% of the population according to the normal distribution of IQ scores). The children belonging to the third profile are likely not to be as rare, and could have difficulties in different domains, although no full-blown disorders, alongside considerable skills.

This new approach provides a view of development that can integrate existing intelligence models such as the Cattell-Horn-Carroll model (CHC Model), revised in particular by McGrew (McGrew and Wendling, 2010; Schneider and McGrew, 2012), Gardner's multiple intelligence model (Gardner, 2003) or Sternberg's “triarchic” model (Sternberg, 1985). The CHC is a hierarchical intelligence model that combines part of the Cattell and Horn model of fluid intelligence (Gf) and crystallized intelligence (Gc) (Cattell, 1941; Horn and Cattell, 1966) with Carroll's “three stratum theory” (Carroll, 1993). This is currently the dominant model, and combines unitarian and pluralist conceptions of intelligence. Carroll evidenced a hierarchical structure comprising three strata: (1) around 40 narrow, highly specific factors; (2) 8 broader factors grouping the 40 factors in the first stratum; (3) a general factor (g-factor). The eight factors of the second stratum are as follows: Fluid Intelligence (Gf), Crystallized Intelligence (Gc), General Memory and Learning (Gy), Broad Visual Perception (Gv), Broad Auditory Perception (Ga), Broad Retrieval Ability (Gr), Broad Cognitive Speediness (Gs), Processing Speed/RT Decision Speed (Gt). The work by Schneider and McGrew (2012) enabled the CHC Model to be completed by adding other factors to stratum 2: Domain-specific knowledge (Gkn), Psychomotor ability (Gp), Psychomotor speed (Gps), Tactile processing (Gh), Kinesthetic processing (Gk), and olfactory processing (Go). The relative consensus provided by the CHC Model strongly influenced the design of the psychometric tests, in particular the Wechsler Scales where FSIQ relates to a general intelligence factor (g-factor) that is still the subject of debate. It can however be noted that, like the Wechsler scales, the CHC Model mainly focuses on purely cognitive functions, and does not take into account the whole range of developmental fields.

The revision produced by Schneider and McGrew (CHC Model v2.1) (Schneider and McGrew, 2012) integrates the psychomotor and sensory dimensions, in addition to the visual and auditory dimensions, but sets aside the socio-emotional field which appears to be composed of more elementary units and could also belong to the 2nd stratum. Over the past decades, there has been research that shows that the socio-emotional field could be part of general development, because it occurs in stages that are ontologically determined. According to these studies, the young child progresses from the “emotional contagion” stage, a state in which it is difficult for him to differentiate himself from others and thus identify an emotion as being that of another person, (for instance an infant that cries at the sound of another infant's crying) (Simner, 1971; Sagi and Hoffman, 1976), toward an “empathy” stage properly speaking (non-synonymous here with “emotional contagion”), a state in which the distinction between self and other enables the developing child to distance emotions belonging to another person, and to protect himself from emotional overwhelming (Hoffman, 1975, 1977; Strayer, 1993; Favre et al., 2009). This trajectory from “emotional contagion” to “empathy” also enables the elaboration of a theory of mind. The socio-emotional sphere appears to be neglected by the current dominant models, despite the fact that it is arousing increasing interest, as can be seen from the work by Gardner, which integrates intra- and inter-personal intelligence, or the work by Sternberg (2000). In all events, it appears essential in the area of the developmental characteristics of children with ASD who present a deficit that is linked to this field of development. This is also true for some children with HIP, sometimes considered to be “emotionally immature.”

This hypothesis for general development profiles affords new perspectives concerning the g-factor, still under debate. Indeed, it appears that the g-factor is relevant within a typical, harmonious developmental trajectory. It however appears to lose significance, like the FSIQ for heterogeneous cognitive profiles, when the developmental pattern is characterized by very variable performances, and independent evolution of the different areas of development.

## Conclusion

In summary, the conclusions of several studies conducted in the field of ASD on the one hand and in the field of HIP on the other hand show some interesting “points of convergence” which could constitute an opening for future research. Indeed, some children with HIP seem to share various clinical signs with children with ASD (sensory atypicalities, attention switching problems, difficulties in emotional regulation), and also cognitive features, more specifically with children with AS (VIQ higher than PIQ; weak PSI, significant IQ discrepancy). From a developmental perspective, a significant IQ discrepancy, often observed in both children with HIP and with ASD, could reflect a heterogeneous developmental pattern according to several studies. Finally, some commonalities might also exist on a neurobiological level (exposure to high levels of fetal testosterone, inter-hemispheric equipotentiality, atypical neurological lateralization, lesser Planum Temporale asymmetry) and a genetic link could be explored with respect to the conclusions of the research by Ruthsatz et al.

The lack of empirical studies designed to compare these populations prevents a thorough analysis, but a new line of investigation can be suggested. It appears that the similarities observed between certain children with HIP and those with ASD without language delays could be explained by the fact that a large proportion of children identified as HIP present a general profile of heterogeneous development, characterized by a heterochronous evolution of the different areas of development, placing them under the heading “developmental disorders” when the deficits are marked. Indeed, some of these children with HIP could present a pattern of strengths and weaknesses similar to children with ASD without language delay. The key features of this developmental pattern remain undetermined, but children with ASD and some with HIP could for example present a developmental delay in specific components of the socio-emotional field. Thus, these children with HIP meet the DSM-5 criteria for ASD in a very moderate or atypical manner, and could be considered as belonging to the “Broad Autism Phenotype.” From a neurobiological point of view, and according to Mrazik and Dombrowski (2010), a prolonged exposure to fetal testosterone, which disorganizes cerebral development, could also constitute a common factor in the development of ASD and HIP.

Beyond clinical features and behavioral manifestations, there seems to be commonalities on more fundamental levels between ASD and HIP, but this question remains complex. This first tenuous link needs to be supported by empirical results from standardized protocols enabling direct comparisons between these populations. More precisely, future research should focus on identifying potential different phenotypes of HIP, or propose a methodology that integrates a distinction between “heterogeneous HIP” and “homogeneous HIP,” not solely on the basis of an IQ assessment but on the basis of a complete neurodevelopmental evaluation including socio-emotional and psychomotor assessments. This first step seems to be essential for a better understanding of what underpins these clinical similarities between a subset of children with HIP and children with ASD. In addition, a comparative study of these specific populations could deepen our understanding of the nature of ASD.

## Author contributions

AB, PP, and LV elaborated the conception, initiated and co-coordinated the writing of the manuscript. AB searched and summarized the literature, wrote the manuscript and created the figures, under the supervision of PP and LV. CH and CD provided their expertise in their respective fields of competence (neuropsychology and genetic psychiatric disorders) and gave feedback on the elaboration of the manuscript. All authors have read and approved the final manuscript.

## Funding

The “Vinatier Hospital Center for the detection and management of rare diseases with psychiatric phenotype” (Lyon, France), and the “Initiative Autisme” placed under the aegis of the “Fondation de France” (Paris, France) encouraged this work and support publication fees.

### Conflict of interest statement

The authors declare that the research was conducted in the absence of any commercial or financial relationships that could be construed as a potential conflict of interest.
